# Self-Esteem Modulates the Time Course of Self-Positivity Bias in Explicit Self-Evaluation

**DOI:** 10.1371/journal.pone.0081169

**Published:** 2013-12-05

**Authors:** Hua Zhang, Lili Guan, Mingming Qi, Juan Yang

**Affiliations:** 1 Key Laboratory of Cognition and Personality, Ministry of Education, Southwest University, Chongqing, China; 2 Department of Psychology, Southwest University, Chongqing, China; University of Bologna, Italy

## Abstract

Researchers have suggested that certain individuals may show a self-positivity bias, rating themselves as possessing more positive personality traits than others. Previous evidence has shown that people evaluate self-related information in such a way as to maintain or enhance self-esteem. However, whether self-esteem would modulate the time course of self-positivity bias in explicit self-evaluation has never been explored. In the present study, 21 participants completed the Rosenberg self-esteem scale and then completed a task where they were instructed to indicate to what extent positive/negative traits described themselves. Behavioral data showed that participants endorsed positive traits as higher in self-relevance compared to the negative traits. Further, participants’ self-esteem levels were positively correlated with their self-positivity bias. Electrophysiological data revealed smaller N1 amplitude and larger late positive component (LPC) amplitude to stimuli consistent with the self-positivity bias (positive-high self-relevant stimuli) when compared to stimuli that were inconsistent with the self-positivity bias (positive-low self-relevant stimuli). Moreover, only in individuals with low self-esteem, the latency of P2 was more pronounced in processing stimuli that were consistent with the self-positivity bias (negative-low self-relevant stimuli) than to stimuli that were inconsistent with the self-positivity bias (positive-low self-relevant stimuli). Overall, the present study provides additional support for the view that low self-esteem as a personality variable would affect the early attentional processing.

## Introduction

An overall desire to feel happy, the desire to maintain or enhance self-esteem, defined as “confidence and satisfaction about oneself”, and a reduction in anxiety about the uncertainty associated with future life outcomes, all result in self-positivity bias [1]. An important feature of self-positivity bias is that people tend to evaluate themselves more positively than third-party observers do [2]. In fact, people judge the self as more positive (or less negative) than they do others on a range of dimensions, such as social skills, achievement, or health [2]. This self-positivity-bias has been termed as “better-than-average” effect when traits are concerned [3]. This effect is quite robust and has been obtained across a diverse representation of samples varying in age, gender, psychopathology, and culture [4], [5].

Self-esteem maintenance was an important reason for self-positivity bias [1]. High self-esteem is characterized by a general fondness and love for oneself, whereas low self-esteem is associated with mildly positive or ambivalent feelings toward oneself [6], [7]. Self-esteem has been linked to a general self-enhancement bias derived from self-ratings of traits representing the Five Factor Model (FFM) [8]. High self-esteem has been found to be related positively to perceiving the self as better than average on both communal and agentic traits [9]. The tendency for individuals to evaluate the self in more favorable terms than they evaluated people in general was particularly pronounced among those with high self-esteem [10].

Functional magnetic resonance imaging (fMRI) results have shown that the medial prefrontal (ventral and dorsal) and medial parietal/posterior cingulate (anterior and posterior) cortices are engaged during tasks which require making specific judgments about one’s own traits compared to judgments of others or semantic judgments [11], [12], [13], [14], [15], while adjacent ventral anterior cingulated cortex (vACC) was able to distinguish emotional valence of this material [16]. Modulation effect of self-esteem on self-positivity bias was also investigated in female participants and results showed that response to valenced self-relevant material within dorsal and ventral medial prefrontal cortex (dmPFC and vmPFC), cingulate cortex, and left temporoparietal cortex varied with individual differences in self-esteem proxy measures [17].

The present study was designed to explore the modulation effects of self-esteem on the time course of self-positivity bias in explicit self-evaluation. The modulation of early components (N1 and P2) by self-relevance was shown in an event-related potential (ERP) study on emotion and self-relevance, suggesting that a self-relevant context can lead to top-down attentional effects during early stages of visual processing [18]. Specifically, anterior N1 has been found to be smaller for “self” stimuli than “other” stimuli, which might reflect a more general influence of self-relevance and lead to top-down attentional amplification of early stages of visual word processing [18]. Therefore, in the present study, we hypothesize that the amplitude of anterior N1 component would be smaller for stimuli that were consistent with the self-positivity bias compared to stimuli that were inconsistent with self-positivity bias. Further, the neural correlates of implicit self-relevant processing in individuals with low self-esteem was investigated and the results showed that self-relevant word processing elicited significantly prolonged peak latency of P2 component to non-self-relevant word processing in low self-esteem [19]. Therefore, we would hypothesize that the latency of anterior P2 would be more pronounced in processing stimuli that were consistent with the self-positivity bias compared to stimuli that were inconsistent with the self-positivity bias in individuals with low self-esteem. Lastly, in a study that examined the time course of self-positivity bias, authors observed, from 450 to 600 ms, a more positive amplitudes to trait adjectives that were consistent with the self-positive bias compared to those that were inconsistent with the self-positivity bias [20], [21]. Therefore, we would hypothesize that the late positive component (LPC) at the posterior location would be larger for adjectives consistent with the self-positivity bias compared to adjectives that were inconsistent with the self-positivity bias. At the behavioral analysis level, we would hypothesize that participants’ self-esteem would be positively correlated with their self-positivity bias. Specifically, high self-esteem people would be more likely to endorse positive adjectives as high self-descriptive and negative adjectives as low in self-relevance.

## Experimental Procedures

### Ethics Statement

The research was approved by Southwest University ethics review board. After participants were given a complete explanation of the study, written informed consent was obtained from all of them.

### Participants

Thirty-two right-handed, healthy university students (18 males, mean age = 21.4 years, SD = 1.9) enrolled at Southwest University, China, participated in the study. For the behavioral data analysis, data from all participants were included. In the electrophysiological data analysis, data from four participants were excluded from analysis because their Beck depression inventory (BDI) scores were higher than 14. Data from seven participants were excluded from analysis because their trials were less than 30 after the data had been preprocessed. Results from the remaining twenty-one participants (14 males, mean age = 21.6 years, SD = 2) were further subdivided into high self-esteem group (8 males, 3 females, mean age = 21.2 years, SD = 2) and low self-esteem group (6 males, 4 females, mean age = 22.1 years, SD = 1.9) based on the mean split of the Rosenberg self-esteem scale scores. The scores for the Rosenberg self-esteem scale were significantly different between the high self-esteem group and low self-esteem group, *F* (1, 19) = 24.63, *p*<0.001; however, neither sex (*p* = 0.56) nor age (*p* = 0.26) were significantly different between groups.

All participants reported no history of or currently suffered from neurological or psychiatric disorder, significant physical illness, head injury, or alcohol/drug abuse (subject self-report). All participants had normal or corrected-to-normal vision. The data and results described in this manuscript were obtained in compliance with the guidelines of APA requirements. The study was approved by the local review board for human participant research and each participant gave their written informed consent prior to participation and was paid for completing the study.

### Materials

Participants completed the Beck depression Inventory (BDI) and the Rosenberg self-esteem scale (RSE). The BDI is one of the most widely used instruments for measuring the severity of depression [22]. The BDI is a 21-question multiple choice self-report inventory, with each answer being scored on a scale value of 0 to 3. Higher total scores indicate more severe depressive symptoms. Cronbach’s α for the BDI is 0.856 in this sample. The RSE is a questionnaire that assesses a person’s overall evaluation of his or her self-worth [23]. The RSE is made up of 10 items such as ‘On the whole, I am satisfied with myself’ or ‘I feel I do not have much to be proud of’ and is coded on a 4-point scale ranging from 1 (strongly disagree) to 4 (strongly agree), with the negative items needing to be reverse scored. Cronbach’s α for the RSE is 0.813 in this sample.

### Procedure

The ERPs were recorded while participants viewed 460 personality-trait adjectives, half of which were positive and half of which were negative. These adjectives were preselected based on the ratings from a previous study [24]. They were instructed to indicate their response, by answering the question “How much does this adjective describe me” using the scale 1 (not at all like me) through 4 (most like me). For each trial, a fixation sign appeared at the center of the screen for 300–500 ms (durations were varied randomly), and then adjectives were presented for 2 s each. Participants were required to press the appropriate keys to indicate their responses as soon as the adjectives appeared on the screen. For further analysis, items attracting a response of 1 or 2 were considered low in self-relevance, whereas items attracting a response of 3 or 4 were considered high in self-relevance.

### Electrophysiological Recording and Analysis

Brain electrical activity was recorded at 64 scalp sites using tin electrodes mounted in an elastic cap (Brain Products, Germany). The cap was placed on the scalp according to the 10–20 system positions with the reference on the left and right mastoids and re-referenced to the average mastoid during post-processing. Vertical and horizontal electrooculogram (EOG) were recorded from above and below the right eye and at the right and left outer canthi, respectively. The inter-electrode impedance was maintained below 5 kΩ at all times. The electroencephalogram (EEG) and EOG were amplified using a 0.05–100 Hz bandpass, and continuous sampling was conducted at 500 Hz/channel during on-line recording.

We used the Brain Vision Analyzer 1.05 software (Brain Products, Germany) for data analysis. Eye movement artifacts (blinks and other movements) were rejected using a Gratton & Coles-based algorithm off-line [25]. Trials with artifacts due to amplifier clipping, bursts of electromyographic (EMG) activity, or peak-to-peak deflection exceeding 80 µV were excluded from averaging. Following these quality control procedures, there were 130 trials left in the negative-low self-relevance condition, 60 trials left for the negative-high self-relevance condition, 35 trials left for the positive-low self-relevance condition and 150 trials left for the positive-high self-relevance condition. A pre-stimulus period of 200 ms was subtracted as a baseline. The amplitudes at Fz, Fcz, Cz, CPz, and Pz were computed on the basis of the signals obtained across cortical midline. The EEG was segmented to obtain epochs extending from 200 ms before to 1000 ms after the stimulus onset. In order to correct for the effect of trial numbers in different condition, the bias correction factor was subtracted from the averaged ERP waveform in each condition for each participants [26], [27]. Then, repeated measures analyses of variance (ANOVA) were conducted on the peak latency and amplitude of N1 (70–150 ms); peak latency and amplitude of P2 (120–300 ms); and mean amplitudes during the time window of 300–500 ms, 500–700 ms, and 700–1000 ms using the following factors as repeated factors: electrode location (Fz, FCz, Cz, CPz, and Pz), valence (positive vs. negative), self-relevance (low vs. high) and group (low self-esteem and high self-esteem). The electrode location, valence, and self-relevance were within-subjects factors and group was included as a between-subjects factor. The *p*-value for all the analyses was corrected for deviations according to the Greenhouse-Geisser.

## Results

### Behavioral Data

Participants’ self-relevance scores and their reaction times for positive and negative adjectives are listed in Table 1. One way ANOVAs were used to assess the effect of valence (positive vs. negative) of adjectives both on reaction time and on self-relevance judgments. For the reaction time, results revealed no significant main effect. For the judgments, results revealed a main effect of valence, *F*(1,31) = 11.8, *p*<0.01, ηp2 = 0.82. Participants’ self-relevance scores were higher for positive traits than for negative traits.

**Table 1:** Participants’ self-relevance scores and reaction times for positive traits and negative traits (32 samples, means and standard deviations).

	Positive traits	Negative traits
**Self-relevance**	2.98(0.21)	2.07(0.34)
**RT**	959(173)	944(174)

Participants’ self-relevance judgment for negative adjectives was then reverse-scored, resulting in a composite measure of self-positivity score for each participant. Correlational analysis revealed that participants’ self-esteem was positively correlated with their self-positivity score, *r* = 0.56, *p*<0.01.

### Electrophysiological Scalp Data

**Figure 1:**
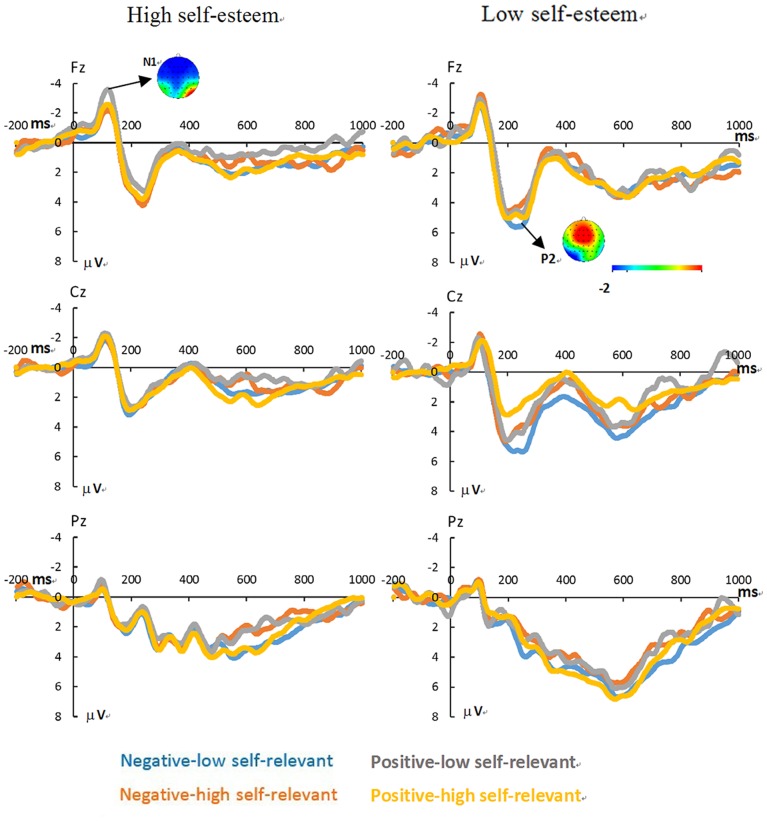
The grand average ERPs for the negative-low self-relevant processing (blue line), the negative-high self-relevant processing (red line), the positive-low self-relevant processing (grey line), and the positive-high self-relevant processing (yellow line) in the low self-esteem group and in the high self-esteem group. The topographic maps of the frontal N1 component in the positive-low self-relevant condition and the frontal P2 component in the negative-low self-relevant condition at electrode of Fz are also shown in Figure 1.

**Figure 2:**
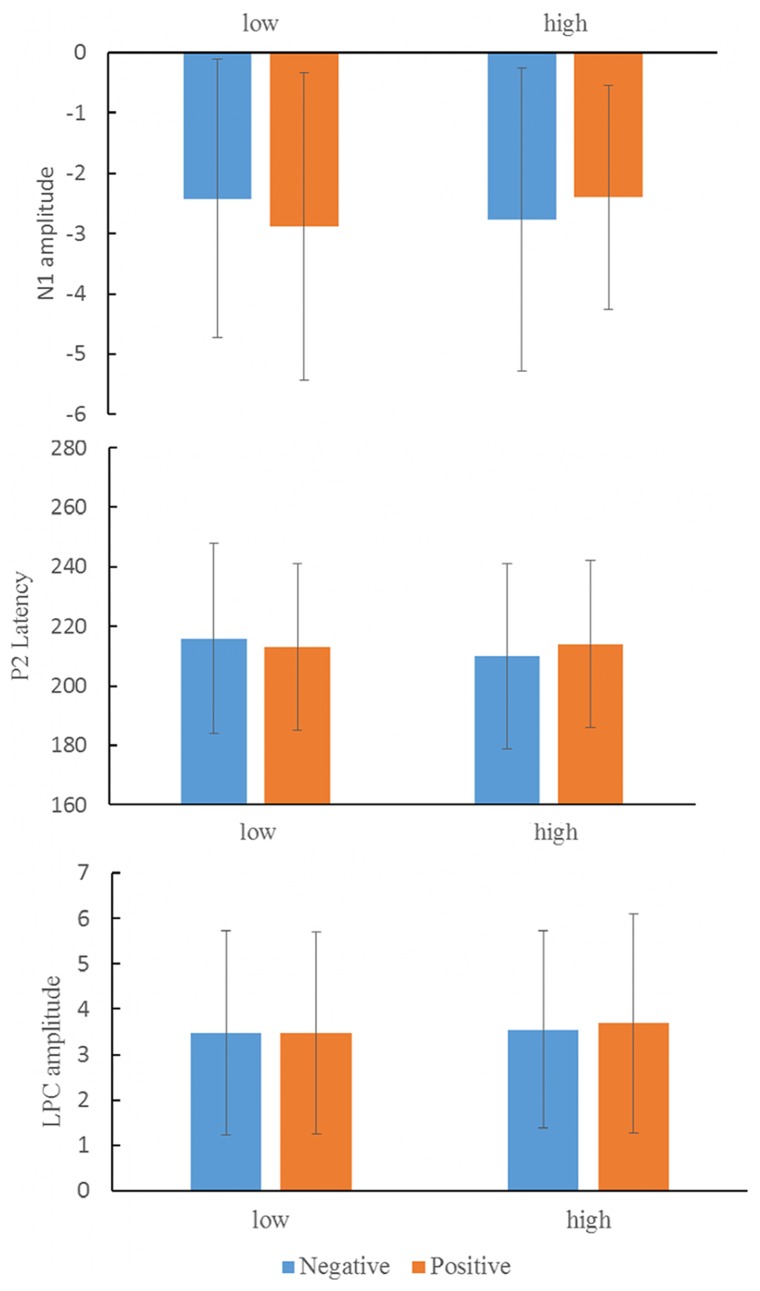
The N1 amplitudes (Top), the latencies of P2 component (Middle) and the LPC amplitudes (Bottom) for positive-high self-relevant words, for positive-low self-relevant words, for negative-high self-relevant words, and for negative-low self-relevant words. Error bars indicate standard deviation of the mean.

The grand average ERPs of the negative-low self-relevant processing, the negative-high self-relevant processing, the positive-low self-relevant processing, and the positive-high self-relevant processing in low self-esteem group and in high self-esteem group are shown in Figure 1. The topographic maps of frontal N1 component in the positive-low self-relevant condition and frontal P2 component in the negative-low self-relevant condition at electrode of Fz are also shown in Figure 1. Table 2 shows the results of the repeated measures ANOVAs for each of the time windows examined. The amplitude of N1 component showed a significant main effect of electrode location, *F* (4, 76) = 8.91, *p*<0.01, ηp2 = 0.32. The amplitude was more pronounced at electrode location of Fz than at the other locations, *p*<0.05. The amplitude of N1 component also showed a significant valence × self-relevance interaction, *F* (1, 19) = 4.66, *p*<0.05, ηp2 = 0.19. Further analysis revealed that N1 amplitude for the positive-low self-relevant stimuli was higher than for both the positive-high self-relevant stimuli and the negative-low self-relevant stimuli (Figure 2, top). The P2 latency showed a significant valence × self-relevance × group interaction, *F* (1, 19) = 8.57, *p*<0.01, ηp2 = 0.31. Within-group analyses further revealed that in the low self-esteem participants, the interaction effect of valence × self-relevance was significant, *F* (1, 9) = 8.35, *p*<0.05, ηp2 = 0.57. The latency of P2 was more pronounced for the negative-low self-relevant processing compared to both the negative-high self-relevant processing and the positive-low self-relevant processing (Figure 2, middle). There was neither an interaction effect of valence × self-relevance in the high self-esteem participants nor any group-based significant differences for P2 component. In the time window of 500–700 ms (LPC), the interaction effect of electrode location × valence × self-relevance was significant, *F* (4, 26) = 4.26, *p*<0.05, ηp2 = 0.18. Follow-ups analyses showed that at the electrode location of Pz, the interaction effect of valence × self-relevance was significant, *F* (1, 20) = 3.59, *p* = 0.07, ηp2 = 0.15. The mean amplitude was more negative for the positive-low self-relevant words compared to both the positive-high self-relevant words and the negative-low self-relevant words. However, the LPC amplitude did not show difference between the positive-high self-relevance processing and the negative-low self-relevance processing (Figure 2, bottom).

**Table 2:** Summary of results for the repeated measures ANOVAs for each of the time windows examined.

Time(ms)	Electrodelocation		Valence×self-relevance		Valence×self-relevance×group		Electrode location×valence×self-relevance	
	F	p	F	p	F	p	F	p
**N1 latency**	8.35	0.01	0.32	ns	0.41	ns	0.32	ns
**N1 amplitude**	8.91	0.01	4.66	0.04	0.02	ns	0.47	ns
**P2 latency**	1.79	ns	2.58	ns	8.57	0.009	1.33	ns
**P2 amplitude**	4.22	0.03	0.45	ns	0.00	ns	0.37	ns
**300–500**	1.75	ns	0.11	ns	3.37	ns	1.09	ns
**500–700**	4.07	0.04	0.16	ns	3.5	ns	4.26	0.02
**700–1000**	0.18	ns	4.76	0.04	0.12	ns	3.13	0.03

ns: no significant.

## Discussion

This ERP study investigated whether self-esteem modulates self-positivity bias in explicit self-evaluation. Participants were instructed to indicate how much a positive/negative traits described them. Behavioral data showed that participants’ self-relevance scores were higher for positive traits than for negative traits. Further, their self-esteem was positively correlated with their self-positivity score. Electrophysiological data found smaller N1 amplitude and larger LPC amplitude to stimuli consistent with the self-positivity bias (positive-high self-relevant stimuli) compared to stimuli that were inconsistent with the self-positivity bias (positive-low self-relevant stimuli). The modulation of self-esteem was reflected in the P2 component. The latency of P2 was more pronounced in processing stimuli that were consistent with the self-positivity bias (negative-low self-relevant stimuli) comapred to stimuli that were inconsistent with the self-positivity bias (positive-low self-relevant stimuli), only in individuals with low self-esteem.

Self-positivity bias is one of the most common and robust findings in social psychology [1]. Individuals rate themselves as possessing more positive personality traits and displaying more positive behaviors than an average people. In addition, individuals rate themselves as having less negative personality traits and negative behavioral characteristics than others [28]. The results of the present study are consistent with this notion that participants are more likely to endorse positive adjectives as being highly self-relevant and endorse negative adjectives as being low in self-relevance.

Researchers have argued that all people prefer to feel good rather than bad about themselves and behave in self-enhancing ways to promote self-esteem [5]. High self-esteem has been found to be related positively to perceiving the self as better than average on both communal and agentic traits [9]. The tendency for individuals to evaluate the self in more favorable terms than they evaluated people in general was particularly pronounced among those with high self-esteem [10]. Correlation result of the present study was consistent with this notion: participants’ self-esteem was positively correlated with their self-positivity score.

Researchers have assumed that the self-positivity bias arises from the motive for self-enhancement [2]. Since most studies of self-enhancement have been conducted in the Western countries, leaving open question of whether people in other cultures, like in East Asia, also self-enhance [2]. On one side of the debate, researchers have suggested that people in Eastern countries, such as Japan, do not show the same self-enhancing tendencies as people in the America [29]. Other researchers have argued that all people behave in self-enhancing ways that promote self-esteem. However, because different characteristics are valued in different cultures, people promote their self-esteem in culturally defined ways [5]. Although the cultural influences are not the main focus of this study, results still indicated that Chinese college participants have a self-positivity-bias in the present study.

Electrophysiological data of the present study showed that the attention-related components, such as the frontal N1 and the frontal P2, were also reflected in the neural basis of self-positivity processing [18]. The N1 is the first negative component elicited 80–130 ms post-visual stimulus onset. The anterior N1, related to focusing attention on task-relevant items prior to perceptual evaluation of a stimulus, reflects different degrees of allocation of attention in response to different stimuli [30], [31]. In an ERP study of emotion and self-relevance, results showed that the prefrontal N1 component was smaller for “self” stimuli than “other” stimuli, which might reflect a more general influence of self-relevance and lead to top-down attentional amplification of early stages of visual word processing [18]. In the present study, the amplitude of N1 component was smaller for stimuli that were consistent with the self-positivity bias than for stimuli that were inconsistent with self-positivity bias. It could be further speculated that stimuli that were inconsistent with self-positivity bias allocated more attention resources than stimuli that were consistent with self-positivity bias.

The P2 component observed in this study was more pronounced at the frontal and central sites than at the parietal scalp sites, which fits the classical scalp distribution of P2 that is related to perceptual analysis and attentiona allocation [32], [33]. The peak latency of frontal P2 is taken as an indication of the time required for perceptual analysis, with slower latency to peak P2 reflecting less efficient processing of visual information at a relatively early stage [34], [35]. In the present study, the peak latency of P2 was more pronounced for the negative-low self-relevant stimuli compared to both the negative-high self-relevant stimuli and the positive-low self-relevant stimuli. Our findings imply that processing of negative-low self-relevant stimuli took more time than processing of other stimuli. However, it should be further noted that this P2 effect was only reflected in low self-esteem participants.

Individuals with low levels of self-esteem are often much more attentive to information concerning social rejection than are those with high self-esteem [36]. This sensitivity is believed to stem from the fact that individuals with low self-esteem have often experienced a considerable amount of social rejection during their lives compared to other individuals [37]. In one study, the impact of self-esteem on attentional bias for social rejection cues was investigated and it was also found that the low self-esteem participants had greater P2 amplitudes in response to social rejection cues compared to neutral cues, suggesting that rejections cues attracted more attentional resources from low self-esteem participants than they did from high self-esteem participants [38]. The self-esteem modulation of on frontal P2 effects has also appeared in another study. In that study, the neural correlates of implicit self-relevant processing in low self-esteem was investigated and the results showed that self-relevant word processing elicited significantly prolonged peak latency of P2 component to non-self-relevant word processing in low self-esteem [19]. The results from both the previous study and the present study indicated that low self-esteem as a personality variable affects the early attentional processing.

Later ERP components, such as P300 and LPC, were found to be enhanced when participants are engaged in higher-order cognitive operations related to selective attention and resource allocation [39]. Electrophysiological studies on the neural processing of self-relevant cues have generally supported the view that the P300 is an index of attention to self-relevant stimuli [40]. Studies on brain correlates of self-positivity bias have repeatedly shown that self-positivity adjectives elicit a larger late positive component (LPC) than non-self-positivity adjectives [20]. Even in the absence of explicit instruction, processing of pleasant words compared to neutral or unpleasant words elicited larger LPC over parietal electrodes after stimulus, in particular when words were self-related [21]. The results of present study were consistent with previous results. As Figure 2 illustrates, the LPC amplitude was greater for stimuli consistent with self-positivity bias (positive-high self-relevant) than for stimuli that were inconsistent self-positivity bias (positive-low self-relevant).

It is important to acknowledge several potential limitations of the present study. First, usage of explicit judgments in this study highlights a potential drawback of this approach in which participants are free to simply judge negative stimuli as not self-relevant [18]. Researchers have started to examine the time course of self-positivity bias in an implicit way [21], [41]. Future studies may try to explore the self-esteem modulation of the time course of self-positivity bias in an implicit way. Second, in another study on self-positivity bias, “other-judgments” are often included as an important control condition [42]. The lack of an “other-condition” in the present study does not allow for calculation of the better-than-average effect.

## Conclusion

The current study was designed to investigate the impact of self-esteem on the time course of self-positivity bias in an explicit self-evaluation task. Our results were consistent with our hypothesis and showed that self-esteem modulates the P2 component. Overall, the present study provides additional support for the view that low self-esteem as a personality variable would affect the early attentional processing.
